# ESD Research of SCR Devices under Harsh Environments

**DOI:** 10.3390/ma16186182

**Published:** 2023-09-13

**Authors:** Chien-Chun Lin, Chun-Yu Lin

**Affiliations:** Department of Electrical Engineering, National Taiwan Normal University, Taipei City 106, Taiwan

**Keywords:** electrostatic discharge, relative humidity (RH), silicon-controlled rectifiers

## Abstract

In prior technology, system-level electrostatic discharge (ESD) tests under environment change conditions mainly focused on testing the effect of a high-temperature environment. i.e., the effect on internal circuits of heat generated outside. However, few studies have explored the effect of ambient relative humidity changes on integrated circuits (ICs). Therefore, this study will analyze the performance of various ESD protection components under high ambient temperature and high ambient relative humidity. The ESD protection devices are tested for the ESD robustness of the silicon-controlled rectifiers (SCR) under a harsh environment and the measurement results are discussed and verified in the CMOS process.

## 1. Introduction

With the rapid development of system-on-chip (SOC) technology, it is possible to integrate an entire electronic system into a single chip in an IC system factory. Thus, a single chip becomes a system. It is necessary to pass the component-level ESD test to ensure the reliability of the component after the chip is manufactured in the wafer foundry. Furthermore, the system is assembled into a product before delivery to the consumer [1,2] and must pass the system-level ESD test. The system-level ESD test standard, IEC 61000-4-2, includes the contact discharge mode and air discharge mode [3]. The IEC standard test is a system-level ESD test that simulates the discharge of an individuals charged device to a system in a system end-user environment. The purpose of the system-level test is to ensure that finished products can survive under normal operation, with consideration of the general assumption that the user of the product will not take any ESD precautions to reduce ESD stress on the product. This ensures that the product does not affect the consumer experience in hot regions or high humidity.

**Figure 1:**
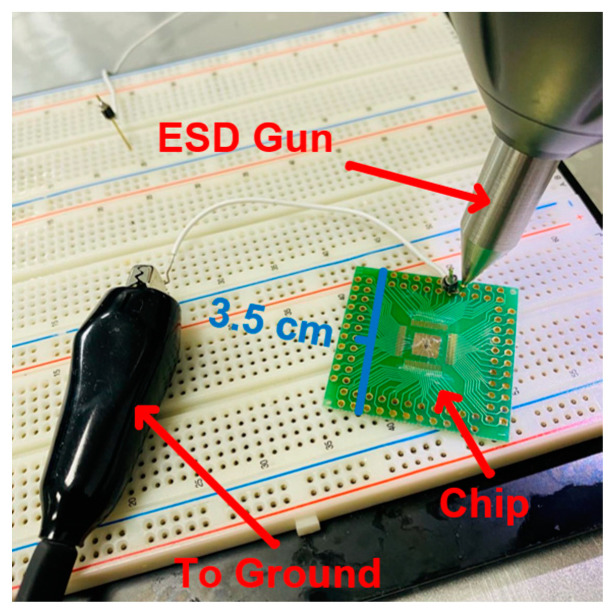
The contact discharge under a system-level ESD test.

ESD events can occur in various settings, such as manufacturing facilities, hospitals, offices, and homes. In manufacturing facilities, ESD events can occur when people handle electronic components or assemble electronic devices. At hospitals, offices, and homes, ESD events can occur when people use medical devices or walk on floors that generate static electricity. Furthermore, ESD failure is the main electronic reliability problem under the system-level and component-level ESD tests. Figure 1 shows that the chip suffered as a result of the firing of an ESD gun under the system-level ESD test. Advanced process technologies will cause the gate oxide layer of the transistor to become thinner and quickly break down under ESD events. In order to avoid ESD events, each input and output terminal in the circuit should be protected by ESD protection circuits [4,5].

ESD is likely to cause damage to automotive electronics under high ambient relative humidity and high ambient temperature. An increase in relative humidity can easily cause a decrease in the ESD robustness of ICs [6]. The temperature of the ICs will increase as the ambient temperature rises. ICs will change their original characteristics due to temperature changes when the temperature of ICs exceed the operating temperature range for which they are adapted [7].

## 2. Typical Design of an On-Chip ESD Protection Component

The use of on-chip ESD protection components is becoming increasingly important as electronic devices become smaller, more complex, and more sensitive to ESD events. Without proper protection, an ESD event can cause irreparable damage to an electronic device, resulting in costly repairs or replacements. On-chip ESD protection components are ICs designed to protect electronic devices and systems from damage caused by ESD stress. These components are typically incorporated into the semiconductor fabrication process to ensure that the final product can withstand ESD events without being permanently damaged. On-chip ESD protection components can take different forms, such as diodes, grounded-gate NMOSs (ggNMOSs), and SCR devices. These ESD protection components are usually used in automotive electronics products and consumer electronics products to protect the operation of internal circuits. After wafer fabrication, a component-level ESD test is required to verify the reliable performance of ESD protection components under ESD events. After the system is assembled into a product and delivered to the consumer, it is necessary to pass the system-level ESD test to ensure that the product will not affect the consumer experience in humidity or in hot areas. This chapter will introduce the working principle of the SCR device and the measurement results under the component-level ESD test and system-level ESD test.

**Figure 2:**
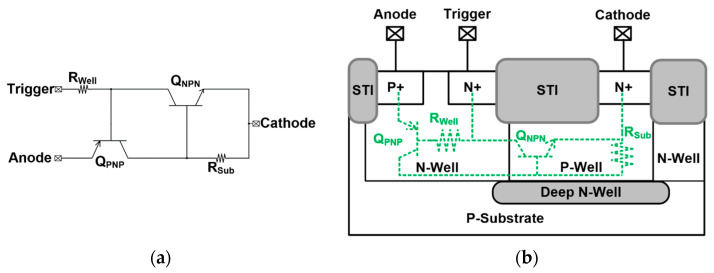
(**a**) Equivalent circuit and (**b**) cross-sectional view of SCR.

To avoid irreversible damage to the chip, which is inflicted via the ESD current from the input terminal, the ESD protection components are needed to protect the internal circuit from ESD current. The SCR device is shown in Figure 2 [8,9]. The SCR device is made of a four-layered P/N/P/N structure, which is equivalent to the cross-coupling structure of the p-n-p BJT and the n-p-n BJT. This cross-coupled structure allows the SCR device to form a positive feedback mechanism. After the ESD is stressed from the anode, the N-Well/P-Well forms the reverse-biased PN junction. When the voltage is reverse biased to the avalanche breakdown point, the SCR device is triggered to discharge the ESD current. It takes time for the SCR device to be triggered, which may cause the internal circuit to be destroyed by ESD. Providing an additional trigger voltage can make the SCR device turn on faster, allowing the SCR device to protect the internal circuit more effectively.

**Figure 3:**
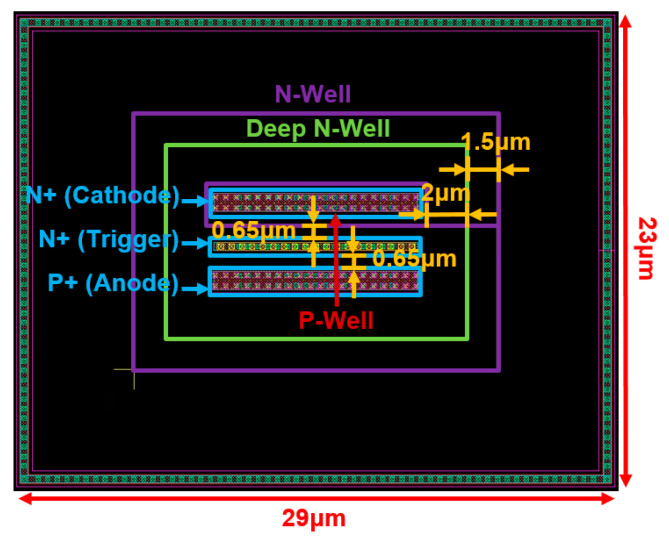
Layout top view of SCR_10μm.

The design parameters of the SCR device are displayed in Table 1. The SCR device is used in the sizes of 10 μm, 25 μm, and 50 μm, namely SCR_10μm, SCR_25μm, and SCR_50μm. Additionally, Figure 3 shows the layout top view of the SCR_10μm.

## 3. Experimental Environment

To ensure that the measurement environment is more accurate, this test uses a humidifier and a heat gun to change the environment. The heat gun can continuously heat the DUT to change the ambient temperature. The operating principle of the heat gun is to use a micro blower as the air source and use an electric heating wire to heat the airflow. The humidifier can continuously humidify the DUT to change the ambient relative humidity. The operating principle of the humidifier is to increase the relative humidity through ultrasonic vibration. The humidification method of ultrasonic vibration can reduce environmental changes, compared with increasing the relative humidity by distributing water vapor through hot water. The temperature will not change with the increase of the relative humidity, and one can then observe the effect of the relative humidity on the ESD protection components.

**Figure 4:**
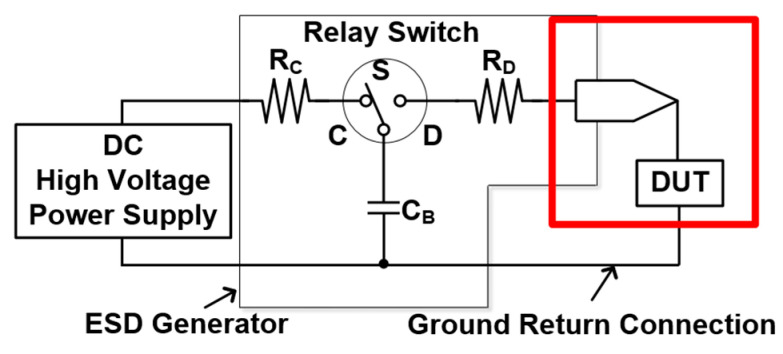
Simplified equivalent circuit of ESD generator in contact discharge mode.

The equivalent circuits of the ESD generator, known as the ESD gun, are shown in Figure 4. The charge resistance (R_C_) is 50~100 MΩ, the discharge resistance (R_D_) is 330 Ω, and the storage capacitance (C_B_) is 150 pF. On the right side of Figure 4, there are two types of discharge methods to the device under test (DUT). One is the contact discharge mode and the other is the air discharge mode. The former is used to measure the effect of the ambient temperature changes on the ESD robustness of the components. The latter is used to measure the effect of the ambient relative humidity changes on the ESD robustness of components. The discharge terminal of the ESD generator is a more intuitive way to judge the mode. For air discharge testing, the terminal must be elliptical, while for contact discharge, the terminal shape is conical. The air discharge testing must have a gap between the ESD gun and the DUT.

**Figure 5:**
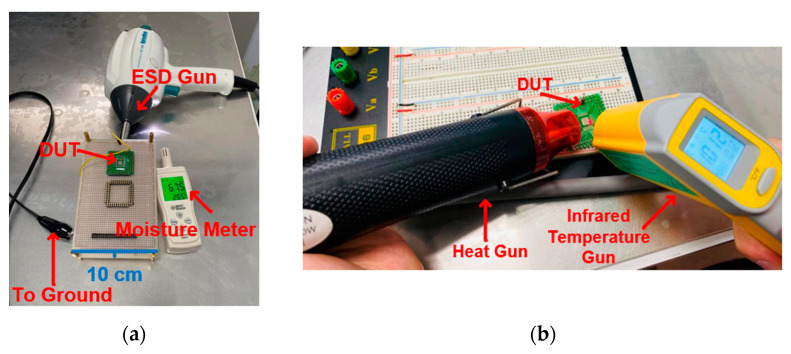
(**a**) Actual measurement of DUT in contact discharge mode and (**b**) schematic diagram of the heating process.

The specific test location is the ESD test table in the laboratory. The heat generated by the heat gun on the metal layer is not easy to dissipate, and the resulting temperature error is small. The measurement equipment of ambient temperature is shown in Figure 5a,b. The DUT can be made to reach 50 °C by continuous heating with the heat gun for 5 s, and it takes 9 s for the ambient temperature to reach 100 °C. The ambient temperature is observed by an infrared temperature gun. When the ambient temperature reaches the desired temperature, the tester will use the ESD gun to conduct the contact discharge on the components. The measurement process will follow the IEC standard test [10,11]. The contact discharge time interval is 1 s, and the contact discharge occurs 3 times. The definition of component damage is damage incurred when the leakage current drifts more than 30% under the system-level ESD test.

**Figure 6:**
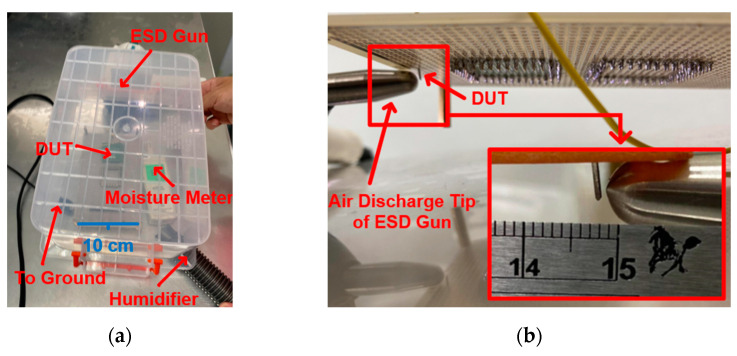
(**a**) Front-side and (**b**) back-side of the measurement environment and DUT in air discharge mode.

The ambient relative humidity is delivered through the plastic box to prevent the moisture from dissipating. The measurement equipment for the ambient relative humidity is shown in Figure 6a. The DUT reaches 70%RH by continuous humidifying for 5 s through the humidifier, and it takes 10 s for the relative humidity to reach 95%RH. The ambient relative humidity is observed by a moisture meter. When the ambient relative humidity reaches the desired relative humidity, the tester will use the ESD gun to perform the air discharge on the components. The air discharge time interval is 2 s, and the air discharge occurs 3 times [12]. The measurement distance between the ESD gun and the DUT is 0.1 cm, as shown in Figure 6b.

The ESD robustness of each device was tested according to IEC 6100-4-2, and the test results were used to determine the discharge voltage. Furthermore, as can be seen in Figure 6, the ESD gun is placed on an ESD test table. The ESD gun does not need to be stabilized by hand. This ensures that the test distance between the ESD gun and the DUT is 0.1 cm.

## 4. Measured Results of ESD Protection Component

**Figure 7:**
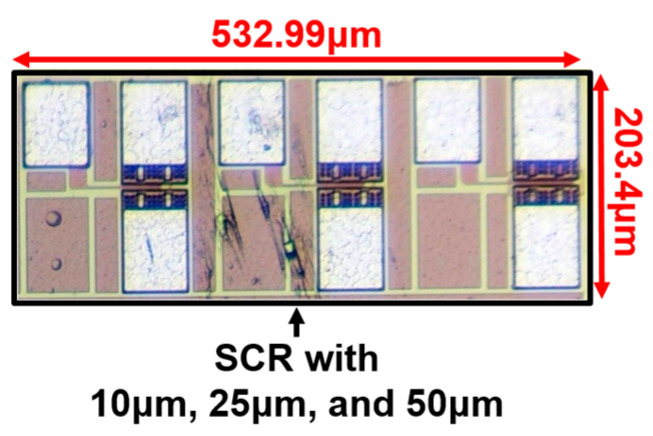
Photograph of ESD protection devices.

The chip photo of ESD protection devices is shown in Figure 7. The total area of ESD protection devices is 532.99 × 203.4 μm^2^. There are three ESD protection devices, including SCR_10μm, SCR_25μm, and SCR_50μm. All devices had been fabricated in the 0.18 μm CMOS process. In this chapter, the measurement results will be discussed. The bare chip had been packaged for testing. Although the outer layer is coated, this does not affect the test results. This work mainly focuses on the impact of ESD zapping on ESD protection circuits after passing through the metal layer. The number of test chips is taken into account. This work used four temperature and three humidity values.

This experiment uses a system-level ESD gun to measure and to understand the ESD robustness of ESD protection devices under ambient temperature and relative humidity. During the measurement process, the ambient relative humidity is controlled at 45%RH, with an increase of 25%RH each time. The effect of humidity on the ESD robustness of components is observed through the changes of ambient relative humidity. When the voltage is at 1.8 V, the leakage current exceeds 30%. This indicates that the DUT has been damaged.

**Figure 8:**
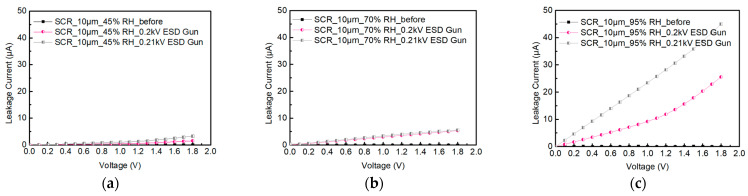
The I–V curves of SCR_10μm before and after system-level ESD zapping at (**a**) 45%RH, (**b**) 70%RH, and (**c**) 95%RH.

During testing SCR_10μm, the ambient relative humidity is humidified from 45%RH to 70%RH and 95%RH. Figure 8a–c shows the system-level ESD robustness of the SCR_10μm. They also show that the leakage current of the SCR_10μm in the air discharge mode exceeds 30% of its original leakage current under different relative humidities. Therefore, the system-level ESD robustness of the SCR_10μm in air discharge mode is less than 0.2 kV, indicating the minimum discharge voltage value of the ESD gun.

**Figure 9:**
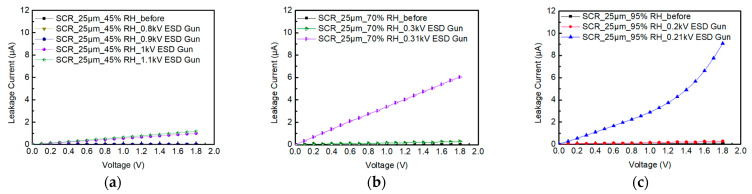
The I–V curves of SCR_25μm before and after system-level ESD zapping at (**a**) 45%RH, (**b**) 70%RH, and (**c**) 95%RH.

During the testing of SCR_25μm, the ambient relative humidity is humidified from 45%RH to 70%RH and to 95%RH. Figure 9 shows the system-level ESD robustness of SCR_25μm. Figure 9a–c also shows that the leakage current of the SCR_25μm exceeds 30% of its original leakage current with the 1 kV, 0.31 kV, and 0.21 kV ESD guns in air discharge mode. The system-level ESD robustness of the SCR_25μm in air discharge mode is 0.9 kV at 45%RH, 0.3 kV at 70%RH, and 0.2 kV at 95%RH. It can be seen that the relative humidity continues to increase, that the system-level ESD robustness of the SCR_25μm will gradually decrease, and that the leakage current will gradually increase.

**Figure 10:**
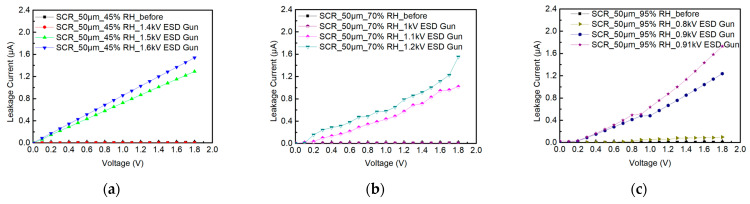
The I–V curves of SCR_50μm before and after system-level ESD zapping at (**a**) 45%RH, (**b**) 70%RH, and (**c**) 95%RH.

During the testing of SCR_50μm, the ambient relative humidity is humidified from 45%RH to 70%RH and to 95%RH. Figure 10 shows the system-level ESD robustness of the SCR_50μm. Figure 10a–c also shows that the leakage current of the SCR_50μm exceeds 30% of its original leakage current with the 1.5 kV, 1.1 kV, and 0.9 kV ESD guns in air discharge mode. The system-level ESD robustness of the SCR_50μm in air discharge mode is 1.4 kV at 45%RH, 1 kV at 70%RH, and 0.8 kV at 95%RH. According to the increase in the leakage current, it can be judged that the system-level ESD robustness of the SCR_50μm will decrease with an increase in relative humidity.

During the measurement process, the ambient temperature was controlled at 25 °C, with an increase of 25 °C each time. The effect of ambient temperature on the ESD robustness of components can be observed under different ambient temperature.

**Figure 11:**
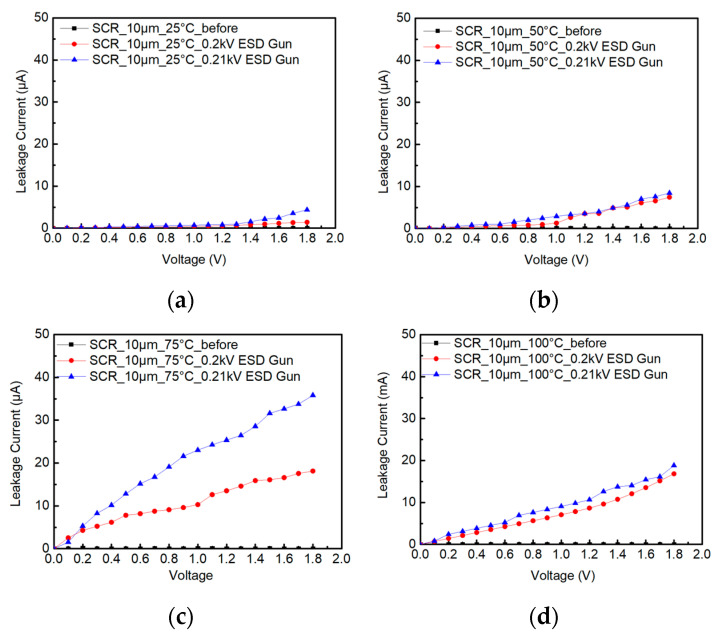
The I–V curves of SCR_10μm before and after system-level ESD zapping at (**a**) 25 °C, (**b**) 50 °C, (**c**) 75 °C, and (**d**) 100 °C.

The ambient temperature is heated from 25 °C to 50 °C, 75 °C and 100 °C during the testing of SCR_10μm. Figure 11a–d shows the system-level ESD robustness of the SCR_10μm. They also show that the leakage current of the SCR_10μm with a 0.2 kV ESD gun in contact discharge mode exceeds 30% of its original leakage current under a different temperature. Therefore, the system-level ESD robustness of the SCR_10μm in contact discharge mode is less than 0.2 kV, indicating the minimum discharge voltage value of the ESD gun. According to the increase in the leakage current, it can be judged that the system-level ESD robustness of the SCR_10μm will decrease with an increase in the ambient temperature.

**Figure 12:**
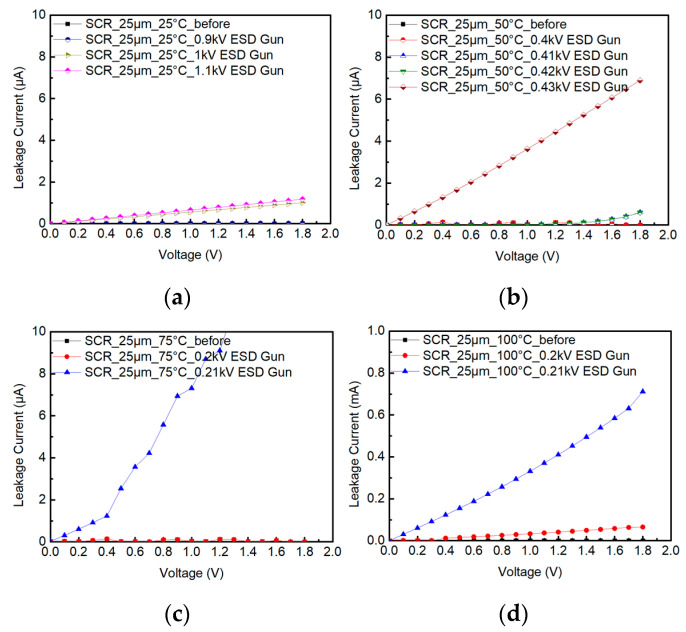
The I–V curves of SCR_25μm before and after system-level ESD zapping at (**a**) 25 °C, (**b**) 50 °C, (**c**) 75 °C, and (**d**) 100 °C.

During the testing of SCR_25μm, the ambient temperature is heated from 25 °C to 50 °C, 75 °C, and 100 °C. Figure 12 shows the system-level ESD robustness of the SCR_25μm. Figure 12a–d also shows that the leakage current of the SCR_25μm exceeds 30% of its original leakage current with the 1 kV, 0.41 kV, 0.21 kV, and 0.2 kV ESD guns in contact discharge mode. The system-level ESD robustness of the SCR_25μm in contact discharge mode is 0.9 kV at 25 °C, 0.4 kV at 50 °C, 0.2 kV at 75 °C, and less than 0.2 kV at 100 °C. According to the increase in the leakage current, it can be seen that the system-level ESD robustness of the SCR_25μm will gradually decrease with an increase in ambient temperature.

**Figure 13:**
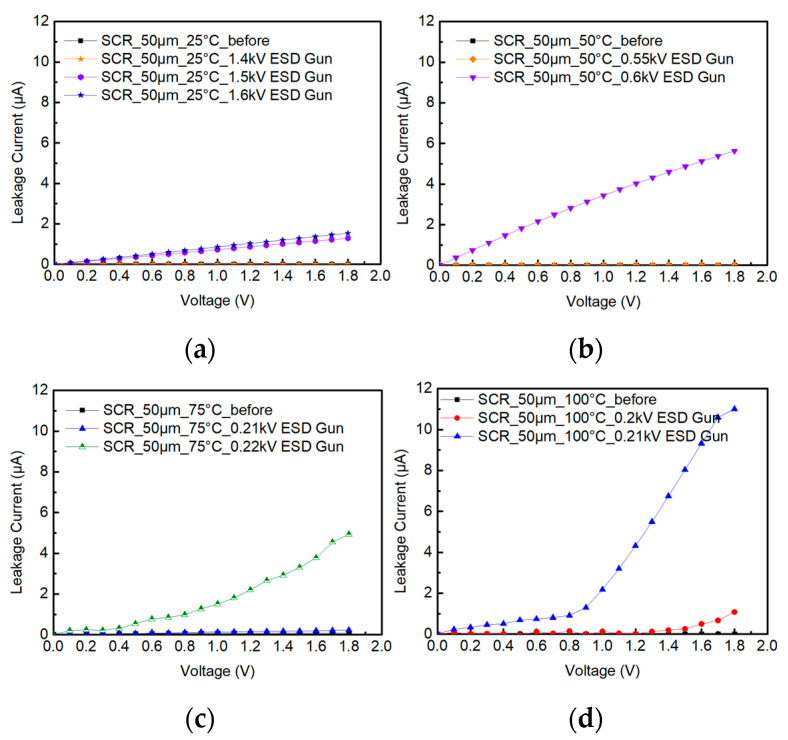
The I–V curves of SCR_50μm before and after system-level ESD zapping at (**a**) 25 °C, (**b**) 50 °C, (**c**) 75 °C, and (**d**) 100 °C.

During the testing of SCR_50μm, the ambient temperature is heated from 25 °C to 50 °C, 75 °C, and 100 °C. Figure 13 shows the system-level ESD robustness of the SCR_50μm, in which the ESD robustness decreases as the temperature increases, and, subsequently, the ESD voltage steps are set to smaller values. Figure 13a–d also shows that the leakage current of the SCR_50μm exceeds 30% of its original leakage current with the 1.5 kV, 0.6 kV, 0.22 kV, and 0.2 kV ESD guns in contact discharge mode. The system-level ESD robustness of the SCR_50μm in contact discharge mode is 1.4 kV at 25 °C, 0.55 kV at 50 °C, 0.21 kV at 75 °C, and less than 0.2 kV at 100 °C. According to the results, it can be seen that the ambient temperature continues to rise, and that the SCR_50μm is more likely to be damaged by the ESD.

**Figure 14:**
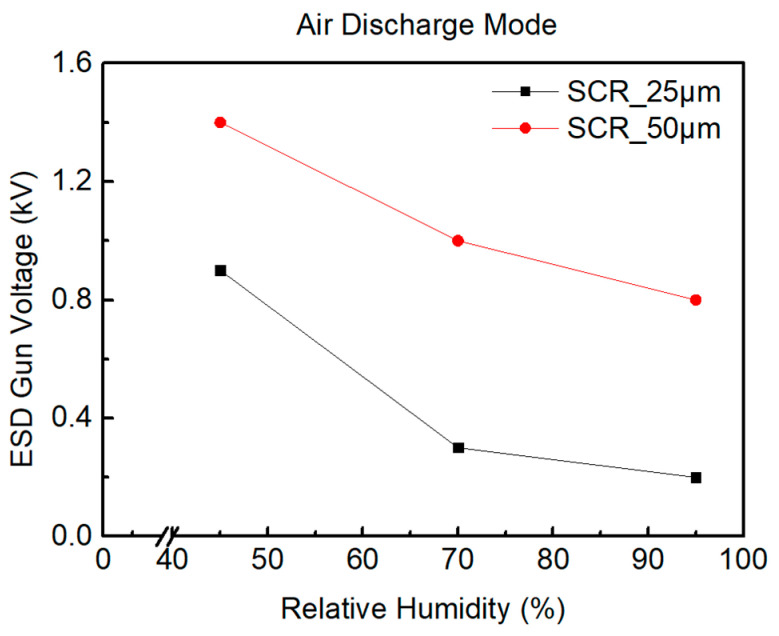
Comparison of system-level ESD robustness of SCR under ambient relative humidity.

According to the measured results, the system-level ESD robustness of the SCR device will decrease with an increase in the ambient relative humidity. The system-level ESD robustness of components in air discharge mode is shown in Table 2. The system-level ESD robustness of the SCR device decreases with increasing relative humidity. An increase in the size of the SCR device increases ESD robustness. The high relative humidity of the environment causes the system-level ESD robustness of the SCR device to decrease significantly under the system-level ESD test, as shown in Figure 14. There are three possible reasons. The first reason is that, in an environment with high relative humidity over a long time, it is easy to form a layer of water on the surface of the components in the ICs [13]. The surface tension in this layer of water will shorten the air discharge distance between the ESD gun and the DUT to a certain extent. This leads to a decrease in the system-level ESD robustness of the SCR device. The second reason is that the surface tension in the water layer is kept as flat as possible [14]. If the distribution of the water layer is not uniform, this will reduce the air discharge distance between the ESD gun and the DUT. This leads to a decrease in the system-level ESD robustness of the SCR device. The third reason is that the dielectric strength of air may decrease under high ambient relative humidity [15]. The dielectric strength refers to the maximum electric field strength that an insulator can withstand without electrical breakdown and also maintains its non-conductive properties. The electrical breakdown occurs when the electric field strength is higher than the dielectric strength of the insulator. The insulator’s resistance drops dramatically and becomes conductive. For gases, an increase in the ambient relative humidity generally results in a decrease in dielectric strength. This means that the distance for the ESD gun to perform air discharge on the DUT will be shortened because the ions in the water can form a conductive channel. This will also lead to a decrease in the system-level ESD robustness of the SCR device [16].

**Figure 15:**
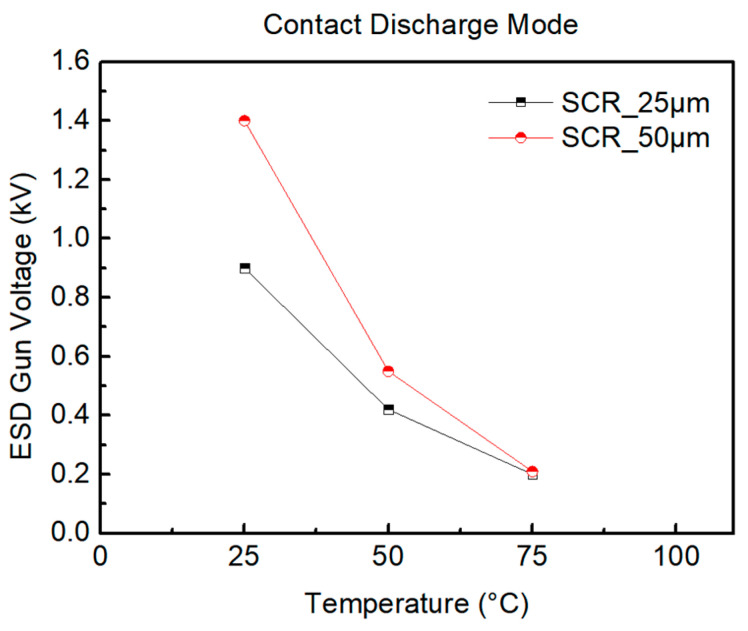
Comparison of system-level ESD robustness of SCR under ambient temperature.

According to the measured results, the system-level ESD robustness of the SCR device will decrease with an increase in the ambient temperature. The system-level ESD robustness of components in contact discharge mode is shown in Table 3. The high ambient temperature causes the system-level ESD robustness of the SCR device to drop significantly, as shown in Figure 15. Compared with the high ambient relative humidity, the changes in the ambient temperature have a significant effect on the system-level ESD robustness of the SCR device. The system-level ESD robustness of the SCR device tends to decrease as the ambient temperature increases. According to the simulated current change of the PN junction formed between N-Well/P-Well under high ambient temperature, it is known that the junction voltage (*V_j_*) will decrease with an increase in ambient temperature. Because the mobility of charge carriers increases with an increase in ambient temperature, the *V_j_* will decrease. According to the ideal diode equation

I_D_ = I_S_ [exp (*qV_j_/kT*) − 1], (1)

the reverse saturation current is I_S_, the size of the basic charge is *q*, the Boltzmann constant is *k*, and the absolute temperature of the junction is *T*. The ESD events are more prone to occur because the higher ambient temperature will increase the mobility of charge carriers. Furthermore, the SCR device can withstand the same power under high ambient temperature and room temperature. It is assumed that the ESD current (I_D_) flowing through the PN junction is a fixed value. The *V_j_* will increase under high ambient temperature. When the cross-voltage of the PN junction increases, the power received by the PN junction will increase and burn it. An increase in the size of the SCR device generally increases its ESD robustness. The SCR device can discharge more ESD current with an increase in size because the parasitic path of the SCR device is widened.

## 5. Conclusions

In this work, non-contact discharge and contact discharge tests were conducted on the different sizes of SCR devices under the ambient relative humidity and temperature. According to the measurement results, the system-level ESD robustness of SCR_10μm is less than 0.2 kV. The actual difference should be measured by a system-level instrument, capable of producing tens of volts. From the SCR_25μm and SCR_50μm, it can be concluded that the component will be subject to an increase in the ambient relative humidity and temperature, which will reduce the system-level ESD tolerance of the ESD protective component.

## Figures and Tables

**Table 1:** Design parameters of SCR.

Test Device	SCR’s Width (μm)	Metal Layers (Anode/Cathode)
SCR_10μm	10	M2~M6/M1~M6
SCR_25μm	25	
SCR_50μm	50	

**Table 2:** Measured system-level results of ESD protection devices under ambient relative humidity.

Test Device	Air Discharge Mode		
	45%RH	70%RH	95%RH
SCR_10μm	<0.2 kV	<0.2 kV	<0.2 kV
SCR_25μm	0.9 kV	0.3 kV	0.2 kV
SCR_50μm	1.4 kV	1 kV	0.8 kV

**Table 3:** Measured system-level results of ESD protection devices under ambient temperature.

Test Device	Contact Discharge Mode			
	25 °C	50 °C	75 °C	100 °C
SCR_10μm	<0.2 kV	<0.2 kV	<0.2 kV	<0.2 kV
SCR_25μm	0.9 kV	0.4 kV	0.2 kV	<0.2 kV
SCR_50μm	1.4 kV	0.55 kV	0.21 kV	<0.2 kV

## Data Availability

Not applicable.
